# Comparative analysis of distinct phenotyping methods for assessing wheat resistance and pathogen virulence among *Fusarium* species causing head blight disease

**DOI:** 10.1186/s13007-025-01402-8

**Published:** 2025-06-16

**Authors:** Vahideh Rafiei, Liza DeGenring, Erin M. Schwister, James Mitch Elmore, Mukesh Dubey, Magnus Karlsson, Milton T. Drott

**Affiliations:** 1 Department of Forest Mycology and Plant Pathology, Uppsala Biocenter, Swedish University of Agricultural Sciences, Box 7026, Uppsala, SE-750 07 Sweden; 2 USDA-ARS Cereal Disease Laboratory, St. Paul, MN USA

**Keywords:** High-throughput phenotyping, Fusarium head blight, Head infection, Cereals

## Abstract

Developing disease-resistant crops is a critical strategy for reducing chemical treatments and mitigating plant disease outbreaks, particularly amid global environmental changes. Fusarium head blight (FHB), caused by a complex of *Fusarium* species, is one of the most devastating cereal diseases, leading to significant economic losses and contamination of grain with harmful mycotoxins that threaten global cereal production and human health. The high variability in virulence within the complex of *Fusarium* spp and the lack of efficient high-throughput screening methods have impeded the development of resistant cultivars and made large-scale virulence testing labor-intensive and time-consuming. This study evaluates the efficacy of detached leaf, coleoptile, and seedling assays as high-throughput alternatives to the standard head infection assay for assessing the virulence of *Fusarium* species and differentiating wheat genotypes by resistance or susceptibility. Two near-isogenic wheat lines, one carrying FHB resistance loci and the other without, were used to assess the virulence of four *Fusarium* species. The seedling and coleoptile assays showed strong concordance with the traditional head infection assay, accurately reflecting differences in disease severity across *Fusarium* species and between wheat lines. Conversely, the detached leaf assay provided some differentiation among species but was inconsistent in identifying differences between plant genotypes. Across all assays, *F. graminearum* consistently exhibited the highest virulence, causing severe disease in leaves, stems, seedlings, and heads, while *F. poae* was the least virulent. Interestingly, *F. culmorum* and *F. avenaceum* displayed tissue-specific variability. These findings establish the coleoptile and seedling assays as rapid, high-throughput alternatives for breeding programs, accelerating the identification of FHB-resistant genotypes and reducing the reliance on the labor-intensive head assay.

**Supplementary Information:**

The online version contains supplementary material available at 10.1186/s13007-025-01402-8.

## Introduction

Extensive research has focused on identifying new sources of resistance to fungal pathogens in cereals [1, 2]. These efforts typically rely on precise assessment of visible phenotypes, such as disease severity and overall plant growth, to aid in the identification of resistant genotypes, map quantitative trait loci (QTLs), and characterize underlying resistance mechanisms [3]. High-throughput phenotyping platforms significantly improve the accuracy of trait measurements, enhancing the selection of superior lines for yield, disease resistance, and stress tolerance [4]. In addition to information on the host-side, these platforms can simultaneously offer insights into the virulence of pathogens under various conditions, allowing for quicker and more informed responses to emerging threats.

Fusarium head blight (FHB), caused by a complex of *Fusarium* species, is a devastating disease that affects wheat (*Triticum aestivum* L.) and other small grains globally [5, 6]. FHB not only reduces yield quality and quantity but also contaminates grain with trichothecene mycotoxins, posing significant health risks to humans and livestock [7]. Several *Fusarium* species, such as *F. avenaceum*, *F. culmorum*, *F. langsethiae*, and *F. poae*, can cause FHB, however, species within the *F. graminearum* species complex (FGSC) are considered the primary cause of FHB epidemics in wheat worldwide [8]. The dominant species responsible for FHB can vary significantly depending on the geographical region and climate conditions. In Europe, *F. graminearum*, *F. culmorum*, *F. poae*, and *F. avenaceum* are the most common species reported in cereal-growing areas [9]. The prevalence of FHB-causing species has recently shifted in many regions and the emergence of some species in new areas is often attributed to changing weather conditions [9–11]. However, agricultural production practices, such as crop rotation, also play a significant role in determining the composition and prevalence of *Fusarium* species in a given region. Crop rotation can affect the persistence of *Fusarium* inoculum in the soil and influence disease pressure by altering host availability and environmental conditions favorable for different species [12–15]. The diversity of species causing FHB makes disease management challenging as different taxa display varying levels of sensitivity to fungicides [16]. An integrated approach that combines resistant cultivars with fungicide applications has been shown to be more effective than using either strategy alone [15]. To address these challenges, high-throughput methods that accurately assess both fungal virulence and host resistance are urgently needed.

Wheat is highly susceptible to FHB in many agricultural regions globally [5]. Although significant progress has been made in understanding resistance mechanisms, complete resistance to FHB has not yet been achieved. Current breeding efforts have focused on incorporating key resistance loci, such as *Fhb1* and *Qfhs.ifa-5 A*, into wheat varieties with desirable agronomic traits [17, 18]. However, breeding FHB resistance in wheat is challenging as resistance is controlled by small- to medium-effect QTLs [19], with variable responses to *Fusarium* infections across growth stages [17–19] Moreover, genetic backgrounds can influence the expression of these QTLs, potentially suppressing the resistance phenotype they mediate [20]. The co-occurrence of multiple *Fusarium* species in cereal fields further complicates disease management [11]. Interactions among species can influence the severity of FHB, levels of mycotoxin contamination, and the dynamics of pathogen spread [21]. Field trials that rely solely on head inoculation methods are widely used and play a critical role in evaluating FHB resistance in wheat. However, these approaches face challenges that can introduce uncertainty in quantifying disease resistance in breeding lines. Their dependence on manual assessments and the need for extensive replications make them time-consuming, costly, and labor-intensive. Additionally, field evaluations are highly prone to variability due to environmental conditions and subjective scoring, which can result in significant experimental errors in disease assessment. While such trials are valuable for assessing general resistance, their ability to evaluate the specific effects of individual *Fusarium* species is limited [22]. Head infection methods can provide insights into *Fusarium* virulence; however, the laborious nature of these assays preclude large-scale factorial experiments aimed at disentangling the complex interactions among coexisting *Fusarium* species that are known to impact FHB disease-control efforts [5, 21].

To address the challenges of traditional phenotyping methods, various high-throughput techniques have been developed to assess *Fusarium* species infection in wheat [23–25]. Comparison of these high-throughput methods to traditional head inoculation shows promise in reducing the reliance on subjective scoring [25]. However, questions remain regarding the ability of these techniques to consistently predict infection stages most relevant to FHB progression and to accurately differentiate between *Fusarium* isolates or wheat genotypes. Previous studies have often focused on a limited number of isolates or wheat cultivars, which may not capture the full complexity of natural infections or genotype-by-environment interactions. Therefore, further validation is required to assess how well these methods perform across a broader range of *Fusarium* species and wheat genotypes. Our study offers insights into high-throughput platforms that can be leveraged to address these gaps. We employ a diverse set of four *Fusarium* isolates and two wheat lines representing resistant and susceptible genotypes, providing a clearer understanding of the strengths and limitations of high-throughput techniques in FHB resistance assessment. While our sample of *Fusarium* isolates and wheat lines is limited to accommodate the testing of multiple methods, the goal of this study was to emphasize the future potential of these methods for pathogen and disease screening.

This study aims to provide deeper insights into the accuracy and reliability of these high-throughput methods for FHB phenotyping, enabling high-throughput standardized screening approaches. Specifically, we aimed to assess high-throughput methodologies for their ability to: (i) identify phenotypic variation in disease symptoms across four *Fusarium* species that are prevalent in cereal-growing areas, (ii) accurately reflect spike responses to FHB infection, and (iii) determine genotype-specific phenotypic responses in wheat using isogenic lines differing in resistance loci. Together, our results provide a clearer understanding of the strengths and limitations of alternative screening techniques for FHB resistance.

## Materials and methods

### Plant materials

The near-isogenic lines (NILs) used in this study were derived from the FHB-resistant wheat genotype ‘CM-82,036’ and the susceptible cultivar ‘Remus’, kindly provided by Dr. Dimitar Douchkov at the Leibniz Institute of Plant Genetics and Crop Plant Research (IPK). The parental line ‘CM-82,036’ originates from a cross between ‘Sumai#3’ and ‘Thornbird-S’, contributes sources of FHB resistance located on QTLs 5 A and 3BS, and exhibits a high level of resistance to FHB comparable to ‘Sumai#3’ [1, 17, 26, 27], while also showing better agronomic traits than Sumai#3. The parental line ‘Remus’, a spring wheat cultivar, originates from the cross ‘Sappo’/‘Mex’/‘Famos’ and is highly susceptible to FHB; however ‘Remus’ possesses well-adapted agronomic traits for cultivation in Europe [1, 28]. The NIL lines combined the desirable agronomic traits of ‘Remus’ but are polymorphic for the FHB resistance QTLs 5A and 3BS from ‘CM-82036’. Specifically, one NIL (3B5A) harbors the resistance loci, while the other NIL (bbaa) does not contain these loci. The presence or absence of these QTLs was described in previous studies, using molecular marker-based selection commonly employed for QTL validation in wheat [1, 17]. These lines were particularly suitable for studying disease symptoms in our experiments due to their enhanced agronomic performance and incorporated resistance sources.

### Fungal isolates and preparation of *fusarium* species inoculum

Four *Fusarium* species, among the most prevalent FHB pathogens worldwide, were used in all assays: *F. graminearum* (PH1), originally isolated from wheat kernels in the USA; and *F. culmorum*, *F. avenaceum*, and *F. poae*, provided by the Department of Ecology, Swedish University of Agricultural Sciences, all originally isolated from wheat crops in Sweden.

The single-spore of each *Fusarium* species were initially cultured on potato-dextrose agar (PDA) for 3–4 days at 25 °C. Macroconidia of the *Fusarium* species were prepared in liquid Mung Bean Broth or Potato Dextrose Broth as previously described [29] and incubated at 25 °C on a horizontal shaker set to 220 rpm for one week. Cultures were subsequently filtered through one layer of sterile Miracloth to collect conidia. Concentrations of conidia were measured and adjusted to 1 × 10^6^ spores ml^-1^ using a hemocytometer and used in all assays except for seed germination assay, where agar plugs were used for inoculation of seeds.

### Experimental design

All phenotyping assays in this study involved five inoculation treatments (four *Fusarium* species and a mock) applied to two wheat genotypes: 3B5A (resistant) and bbaa (susceptible), resulting in a total of 10 treatments. The number of replicates per experiment differed across assays and is detailed below.

### Coleoptile infection assay

The coleoptile assay was conducted following the protocols detailed previously [30] with modifications. Briefly, wheat seeds were washed with sterile water three times and germinated on moist filter paper in Petri dishes. Once the coleoptiles had emerged after 3–4 days of germination, each coleoptile was placed individually into a well of a 24-well cell culture plate, on small pieces of autoclaved paper (Kimtech Science, Kimberly-Clark Professional, Roswell, GA, USA), which were moistened with sterile water (Fig. S1a, b). For inoculation, we removed 1–2 mm from the tips of three-day-old coleoptiles and a 5 µL droplet of spore suspension (10⁶ conidia/mL) was placed on the cut tip of each coleoptile. For mock inoculation, 5 µL of sterile water was added to the wounded tip. Each treatment was applied to 12 replicate coleoptiles, with one coleoptile per well.

The coleoptile assay followed a completely randomized design (CRD) with 12 replicates per treatment. Each coleoptile, representing a replicate, was placed into individual wells of a 24-well plate and randomly assigned a treatment. The plate was placed inside a plastic box and 2 ml of water was added to the bottom of each box to maintain humidity. The box was covered with clear plastic bags to increase humidity and placed in a growth chamber with a 16-hour light/8-hour dark photoperiod at 20 °C for 7 days. Disease symptoms were evaluated by measuring the longitudinal length of brown lesions on the wheat plants’ stems and leaves using a ruler at 7 days post-inoculation (dpi) (Fig. 2a).

### Seedling assay

Seedling assays were performed using the method described previously [31, 32]. Seeds of the NILs were sterilized using 1% NaOH for 5 min with periodic shaking. After sterilization, seeds were washed three times with distilled water prior to inoculation. For each treatment, three wheat seeds were placed in each of 15 pots filled with sterile sand to facilitate seedling harvesting and minimize root damage during assessment. A 3 mm mycelial plug was extracted from fresh fungal colonies (five-day old) of *Fusarium* species, cultivated on PDA, and placed in the middle of the three planted seeds to infect the wheat seedlings. The pots were then incubated for 14 days under a 16-hour light/8-hour dark photoperiod at 20 °C and 65% humidity (Fig. S2a).

Disease assessment included the following parameters: percentage of germination, shoot length, root length, and disease severity. Disease severity was scored on a scale from 0 to 4, where 0 = no symptoms, 1 = slightly brown symptoms on the coleoptile or roots, 2 = obvious brown symptoms on the coleoptile and roots, 3 = severe browning and near death of coleoptile and roots, and 4 = a dead plant [31], (Fig. S2b). This experiment followed a CRD with 15 replicates per treatment, where each replicate was represented by a pot containing three seedlings. The three seedlings within each pot were considered subsamples, and their disease severity was averaged to obtain a single pot value. These pot averages were then used for statistical analysis.

### Leaf assay

The detached leaf assay was performed following the protocol described by Browne and Cooke [23] with adjustments by Perochon and Doohan [33] and further modifications in this study. Briefly, seeds of the NILs were washed three times with sterile water and sown in pots containing 150 g of peat. The plants were grown in a growth chamber with a 16-hour light/8-hour dark photoperiod at 20 °C and 65–70% relative humidity. When seedlings reached the three-leaf stage, the second leaves were harvested for the infection assay. Six uniform leaf segments (6 cm in length) were obtained from the middle part of the harvested leaves and placed on square petri dishes containing 1% water agar with 0.5 mM benzimidazole to inhibit leaf senescence. Before placing the leaves on the petri dishes, the middle parts of the agar plate were removed. These agar pieces were then placed on either end of the leaf segments to fix the leaves in place and prevent excessive growth of *Fusarium* sp. on the agar at the inoculation site. The center of each leaf section was punctured at the midvein with a glass Pasteur pipette and inoculated with a 10 µl droplet of conidial suspension (10⁶ conidia/mL) or sterile water. The plates were covered and incubated in a climate chamber at 20 °C with a 16-hour light/8-hour dark photoperiod.

This assay followed a CRD where each treatment was applied to three Petri dishes each with six leaf segments, for a total of 18 replicates per treatment [33]. Disease development was assessed 3 dpi by measuring the lesion area and total leaf area using Fiji [34]. The percent lesion area was calculated by taking the lesion size, dividing it by the leaf area, and multiplying by 100. To establish homoscedasticity, all percent lesion area data were transformed by adding 0.5 and taking the square root prior to analysis (Fig. 3a, b).

### Head infection assay

The head (spike) infection assay was conducted according to methods described by Mesterházy et al. [35] and Feng et al. [29]. Two infection methods were tested: the first involved applying a droplet of conidial suspension to individual spikelets [35], and the second involved inoculating wheat heads with agar discs (~ 2 mm) taken from fresh *Fusarium* cultures on PDA, commonly used for strains that do not produce conidia [29]. In our study, the agar disc method always resulted in severe symptoms, leading to rapid death of the spikes within a few days (data not shown). Because this method seemed to offer very little resolution between isolates and seems less biologically relevant to field conditions, we proceeded with the conidial suspension method.

Wheat plants were grown under controlled growth chamber conditions (20 °C, 16-hour light/8-hour dark photoperiod). Both wheat genotypes used in this study were spring types and took approximately 10 to 12 weeks from planting to reach the anthesis stage, at which point the spikes were mature enough for infection assays. For each treatment, 8 replicate wheat spikes were inoculated in a CRD, ensuring random placement and numbering of individual spikes within the growth chamber. Spikes were infected by placing a 20 µL conidial suspension (10⁶ conidia/mL) on the floret located between the lemma and palea of the third or fourth spikelet from the base of the spike (Fig. 4a, b). After inoculation, the spikes were covered with plastic bags to maintain humidity for 36 h before removal. Disease symptoms were evaluated 10–12 dpi by assessing the extent of bleaching and necrosis on the spikelets. Disease severity was assessed by counting the number of infected spikelets and rating them on a 0–4 scale (where 0 = no infection, 1 = 25%, 2 = 50%, 3 = 75%, 4 = 100% of the spikelets were bleached) [35]. This scale was used to generate the head infection disease index as a measure of disease severity [35] (Fig. 4c).

### Data analysis

Data analysis was performed using RStudio version 2024.06.05 “Chocolate Cosmos” [36]. Data was analyzed using a factorial analysis of variance (ANOVA), considering wheat genotype, *Fusarium* species, and their interaction as factors. Assumptions of normality of residuals and homogeneity of residuals were tested using the Shapiro-Wilk test and Levene’s Test in R. Post hoc Tukey means separation tests were conducted on treatment means. Post hoc Tukey means separation tests (α < 0.05) were conducted on treatment means Graphs were created in R using the package ‘ggplot2’ (version 3.5.1).

To determine the correlation between high-throughput assays and the canonical head assay, two correlative approaches were taken. First, spearman correlation coefficients and corresponding p-values were calculated using the Spearman rank-order correlation test (cor.test function) on the mean values for each treatment. Second, within each assay measurement, the data were rank normalized using the package ‘dpylr’ (version 1.1.4) to compare measurements across different assays. To have a positive correlation between all measurements, the percent inhibition was calculated for the germination rate, shoot length, and root length for the seedling assay using the mock treatment as the control. Then the effect of assay was analyzed with a whole-model ANOVA and Tukey means separation test.

## Results

### Comparative correlation of tested assays with head assay

All disease index measurements significantly correlated to the head infection disease index (*P* ≤ 1.54E-02) based on the Spearman correlation test (Table 1). As expected, seedling shoot and root lengths were negatively correlated to the head infection disease index (the number of infected spikelets) as they are measurements of plant health, not disease severity (Table 1). Seed germination rate was also negatively correlated to the head infection disease index.

**Table 1:** Spearman correlation coefficients and corresponding p-values for the comparison between head infection disease index measurements and other measurements from the three assays (coleoptile, seedling, and leaf)

Comparison against Head Infection Disease Index	Spearman Correlation Coefficient	*p*-value
Coleoptile stem lesion	0.954	1.76E-05
Coleoptile leaf lesion	0.809	4.58E-03
Seedling germination rate	-0.905	3.14E-04
Seedling shoot length	-0.750	1.24E-02
Seedling root length	-0.856	1.54E-03
Seedling disease index	0.959	1.10E-05
Leaf lesion	0.886	6.37E-04

In the whole-model ANOVA, there was a significant three-way interaction between host genotype, *Fusarium* species, and assay on rank-normalized disease measurements (*P* = 0.013; Table S1). This suggests that the relationship between *Fusarium* species and disease severity depends on the assay type and wheat genotype. Given our focus on high-throughput assays, we further explored the impact of assay type for each *Fusarium* species individually to better understand this interaction. Detailed ANOVA tables can be found in Supplementary Tables 2–6.

There was no significant difference in disease caused by *F. graminearum*, *F. culmorum*, and *F. avenaceum*, between the head, coleoptile, and seedling assays on resistant and susceptible hosts (*P* ≥ 0.108; Table 2). This suggests that the coleoptile and seedling assay are effective high-throughput assays that will yield similar results as the more-laborious head assay across host genotypes and pathogen species. However, for *F. poae*, only the seedling assay was similar to the head assay in terms of rank-normalized disease measurements (*P* = 0.339) (Table 2). *F. poae* consistently caused low levels of disease across all assays, causing no disease at all when inoculated onto heads. However, *F. poae* is known to sometimes cause FHB [5, 7]. Given that the high-throughput methodologies did capture this disease-causing capability, we speculate that these assays may offer more-replicable measures of pathogens causing low disease severity.

Overall, the tested assays show potential for estimating virulence and host resistance across several species, emphasizing the potential for further validation with a larger collection of isolates and wheat lines to establish biological trends observed here between species and hosts. While the predictive power of the assays differs for *F. poae*, this variation appears to be driven by the absence of disease in the head assay, whereas very low disease levels were observed in the other assays. Given the complexity of the three-way interaction, each assay was analyzed separately in subsequent sections to better understand the effects of genotype and *Fusarium* species on disease measurements.

**Table 2:** Mean rank-normalized disease measurements ± standard error for both resistant and susceptible lines across different assays and *Fusarium* species inoculations. Within each assay measurement, the data were rank normalized and then combined to compare measurements across different assays. Within each species inoculation, means followed by the same letter are not significantly different (α = 0.05) as determined by the Tukey HSD post-hoc test

Assay	*F. graminearum*		*F. culmorum*		*F. avenaceum*		*F. poae*		Mock	
Head	82.4 ± 3.8	ab	76.3 ± 3.5	a	64.7 ± 1.7	ab	60.0 ± 0.0	a	60.0 ± 0.0	a
Leaf	90.0 ± 1.0	a	53.3 ± 2.2	b	57.8 ± 3.1	b	38.8 ± 2.7	b	20.2 ± 0.9	c
Coleoptile	80.4 ± 2.4	b	66.1 ± 2.6	a	71.2 ± 2.2	a	41.6 ± 0.8	b	40.0 ± 0.7	b
Seedling	86.5 ± 1.0	a	74.0 ± 1.5	a	57.1 ± 2.0	b	52.2 ± 2.0	a	37.3 ± 2.4	b

### Coleoptile assay

**Fig. 1:**
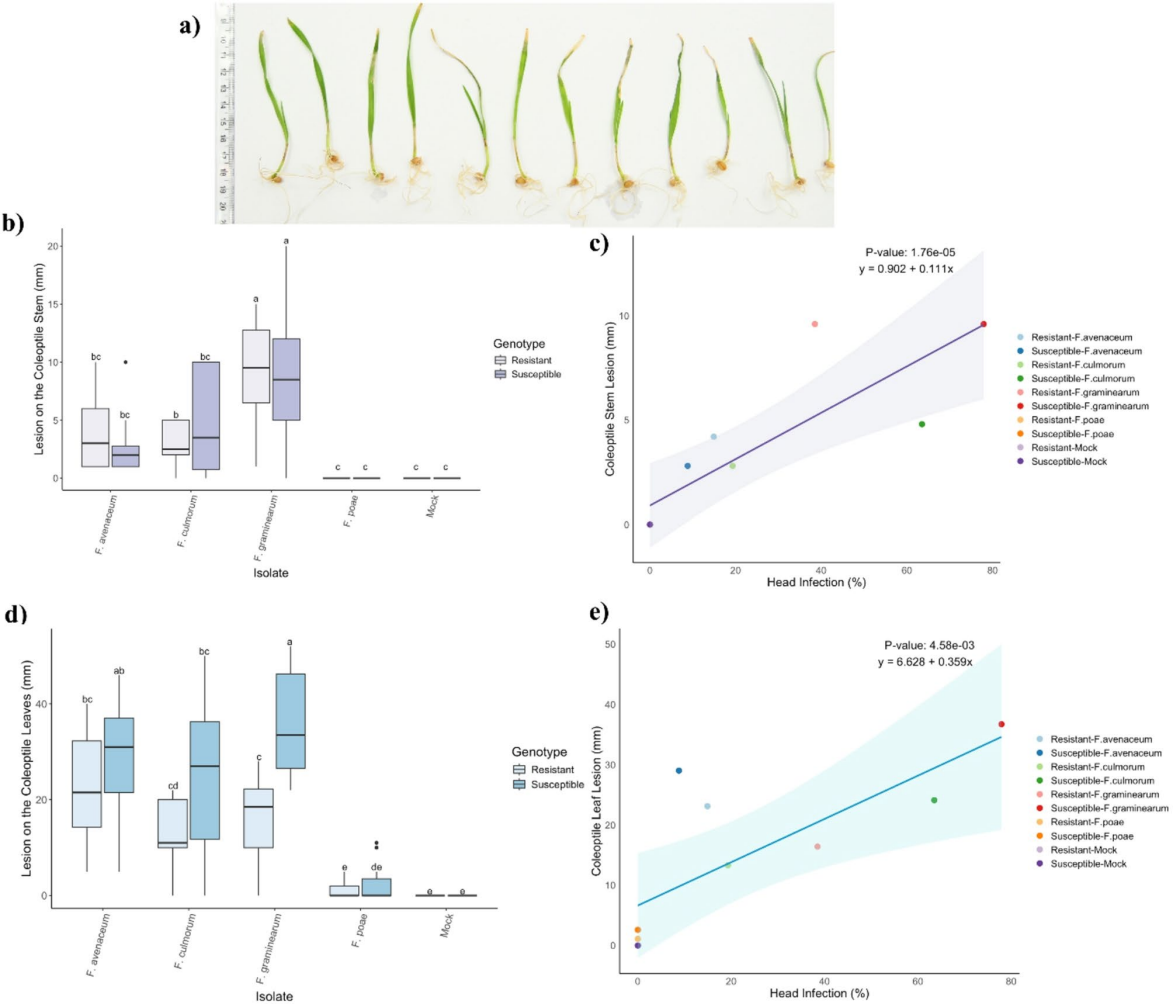
Lesions on the stems and leaves in coleoptile assay were measured 7 days after inoculation. **a)** A representative image from different replicates of the susceptible wheat genotype (bbaa) infected by *F. graminearum*. **b)** Effect of *Fusarium* species treatment on coleoptile stem lesion size (mm) on resistant (3B5A) and susceptible (bbaa) wheat for the coleoptile assay. Different letters above each boxplot indicate significant (α = 0.05) differences in group means as determined by the Tukey HSD Post-hoc test (*n* = 12). **c)** Scatterplot comparing stem lesion size in coleoptile assay to head infection assay. The regression line indicates a significant correlation (*P* = 1.76-05), with the shaded area representing the 95% confidence interval. **d**) Effect of *Fusarium* species treatment on coleoptile leaf lesion size (mm) on resistant (3B5A) and susceptible (bbaa) wheat for the coleoptile assay. **e)** Scatterplot comparing leaf lesion size in coleoptile assay to head infection assay. The regression line indicates a significant correlation (*P* = 4.58E-03), with the shaded area representing the 95% confidence interval

For the coleoptile stem assay, there was no interaction between wheat genotype and *Fusarium* species on stem lesion size (*P* = 0.489; Table S7), indicating that the effect of host resistance was consistent across all species tested. Stem lesion size was not significantly influenced by wheat cultivar (*P* = 0.842), but there was a strong effect of *Fusarium* species on lesion size on stem (*P* < 0.001) (Fig. 1b). Specifically, coleoptiles treated with *F. graminearum* developed the largest stem lesions on average (9.6 mm), nearly twice as large as those caused by other *Fusarium* species. This lesion size represents the mean across both resistant and susceptible wheat genotypes, as no statistically significant differences were observed between them in this assay *F. culmorum* and *F. avenaceum* also caused significantly larger lesions (2.8–4.8 mm) compared to *F. poae* and the mock treatments (*P* ≤ 0.002). Notably, *F. poae* did not produce measurable disease in this assay. These findings suggest that differences in virulence between *Fusarium* species are a major driver of disease severity in the coleoptile stem lesion, regardless of the wheat genotype.

For the coleoptile leaf lesion size (Fig. 1a), there was an interaction between wheat genotype and *Fusarium* species treatments on lesion size (*P* = 0.001; Table S8), thus the simple effects were analyzed. While leaf lesions (Fig. 1a) were similar to those in the stem lesion, the species effect was significant only for wheat lines inoculated with *F. graminearum*, where susceptible wheat genotypes developed significantly larger leaf lesions (36.7 mm) than resistant genotypes (16.4 mm) (*P* < 0.001) (Fig. 1d). Although other *Fusarium* species trended towards a similar pattern, these differences were not statistically significant between wheat lines (*P* ≥ 0.126). Both leaf and stem lesion sizes were positively correlated with head infection results across genotypes (Fig. 1c and e). Overall, both results from coleoptile stem and leaf lesions assays showed that *F. graminearum* is the most virulent species, particularly on susceptible wheat.

### Seedling assays

#### Impact of infection on seedling germination rate

Wheat genotype did not modify the effect of *Fusarium* species on germination rates (*P* = 0.728; Table S9), so the main effects were analyzed. The resistance of wheat did not influence whether the seedlings germinated (*P* = 0.164). The germination assay showed that *F. graminearum*, *F. avenaceum*, and *F. culmorum* caused significant reductions in seed viability compared to *F. poae* and the mock-treated control (*P* = 0.0474; Fig. S3).

#### Impact of infection on shoot and root length

Shoot lengths were significantly different between wheat genotypes depending on the *Fusarium* species inoculated (significant interaction, *P* < 0.001; Table S10), thus simple effects were analyzed. Seedlings treated with *F. graminearum* had the shortest shoots on both host genotypes (*P* < 0.001; Fig. S4a). Although the resistant cultivars generally had shorter shoot lengths across species, this was only significant for seedlings treated with *F. culmorum* and *F. poae* (*P* < 0.001).

There was no significant interaction between wheat genotype and *Fusarium* species on root length (*P* = 0.065; Table S11) thus main effects were analyzed. Seedlings treated with *F. graminearum* and *F. culmorum* exhibited the shortest root lengths, ranging from 9.0 to 11.8 mm (*P* < 0.001; Fig. S4b). For *F. avenaceum* inoculated seedlings, there was a significant effect of genotype, as the resistant cultivar had longer roots (15.6 mm) compared to the susceptible cultivar (13.9 mm) (*P* = 0.013). This finding supports the hypothesis that resistant cultivars (3B5A) maintain better root development even when exposed to pathogens, indicating their potential for improved resilience against *Fusarium* infections.

### Disease severity assessment of seedlings

**Fig. 2:**
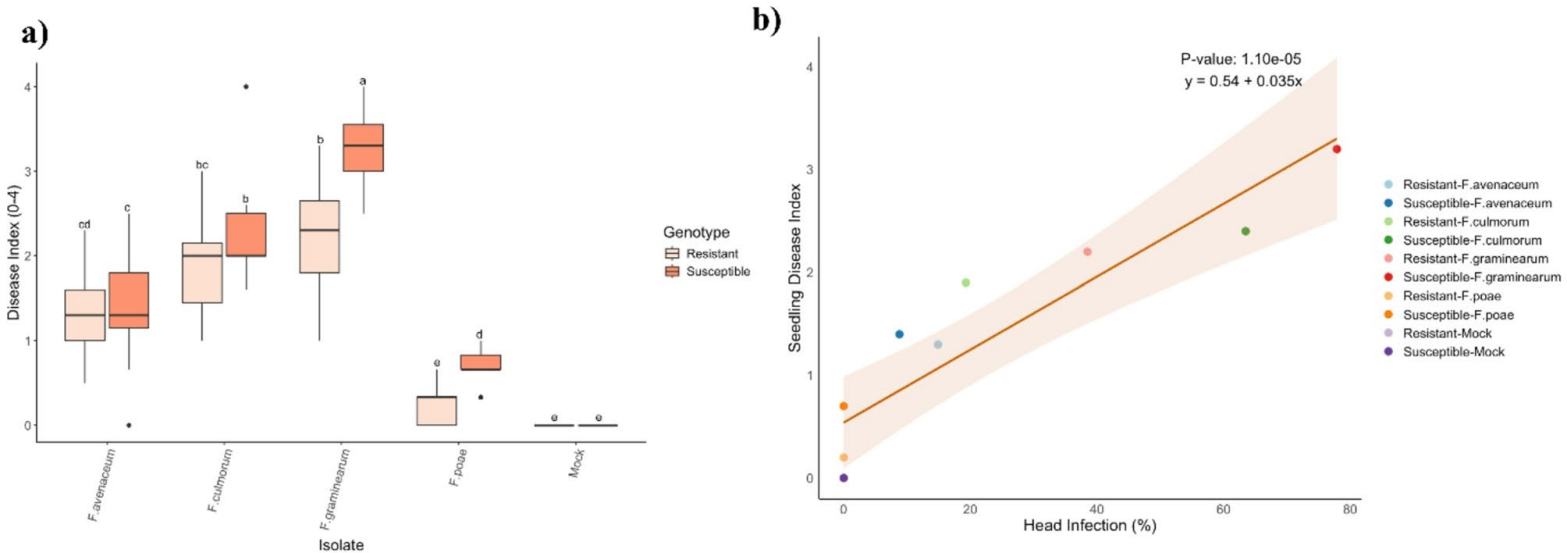
**a)** Effect of *Fusarium* species treatment on disease index (0–4) on resistant (3B5A) and susceptible (bbaa) wheat for the seedling assay 14 days post inoculation. Different letters above each boxplot indicate statistically significant (α = 0.05) differences in group means as determined by the Tukey HSD Post-hoc test (*n* = 15). **b)** Scatterplot comparing seedling disease index in seedling assay to head infection assay. The regression line indicates a significant correlation (*P* = 1.10E-05), with the shaded area representing the 95% confidence interval

The amount of disease caused by *Fusarium* species in the seedling disease assay was different between host genotypes (significant interaction, *P* < 0.001; Table S12), thus the simple effects were analyzed. Resistant wheat genotypes had lower disease indices compared to the susceptible genotypes, but this was only significant for seedlings inoculated with *F. graminearum* and *F. poae* (*P* ≤ 0.002; Fig. 2a). *F. graminearum* and *F. culmorum* caused the highest disease indices (2.2–3.2), while *F. poae* and *F. avenaceum* caused significantly less disease (*P* ≤ 0.006) (Fig. S5a, b). A positive correlation between the seedling disease index and head infection percentage (Fig. 2b) supports the potential utilization of the seedling assay as a high-throughput alternative to the spike-infection assay for disease severity assessments.

The findings from seedling assay revealed significant insights into the interaction between wheat genotypes and various *Fusarium* species, highlighting the critical role of host resistance in mitigating disease severity. Resistant wheat genotypes consistently, although not always statistically significant, demonstrated lower disease indices compared to susceptible ones, particularly when exposed to the aggressive isolate *F. graminearum* (Fig. 2a). Seedlings treated with *F. graminearum* and *F. culmorum* exhibited reduced shoot and root lengths compared to seedlings treated with the other *Fusarium* species, indicating the strong virulence of these species (Fig. S4a, b). Germination rates were not significantly affected by genotypes or species, suggesting that while resistance may not impact germination, it plays a crucial role in post-germination growth (Fig. S3).

### Leaf assay

**Fig. 3:**
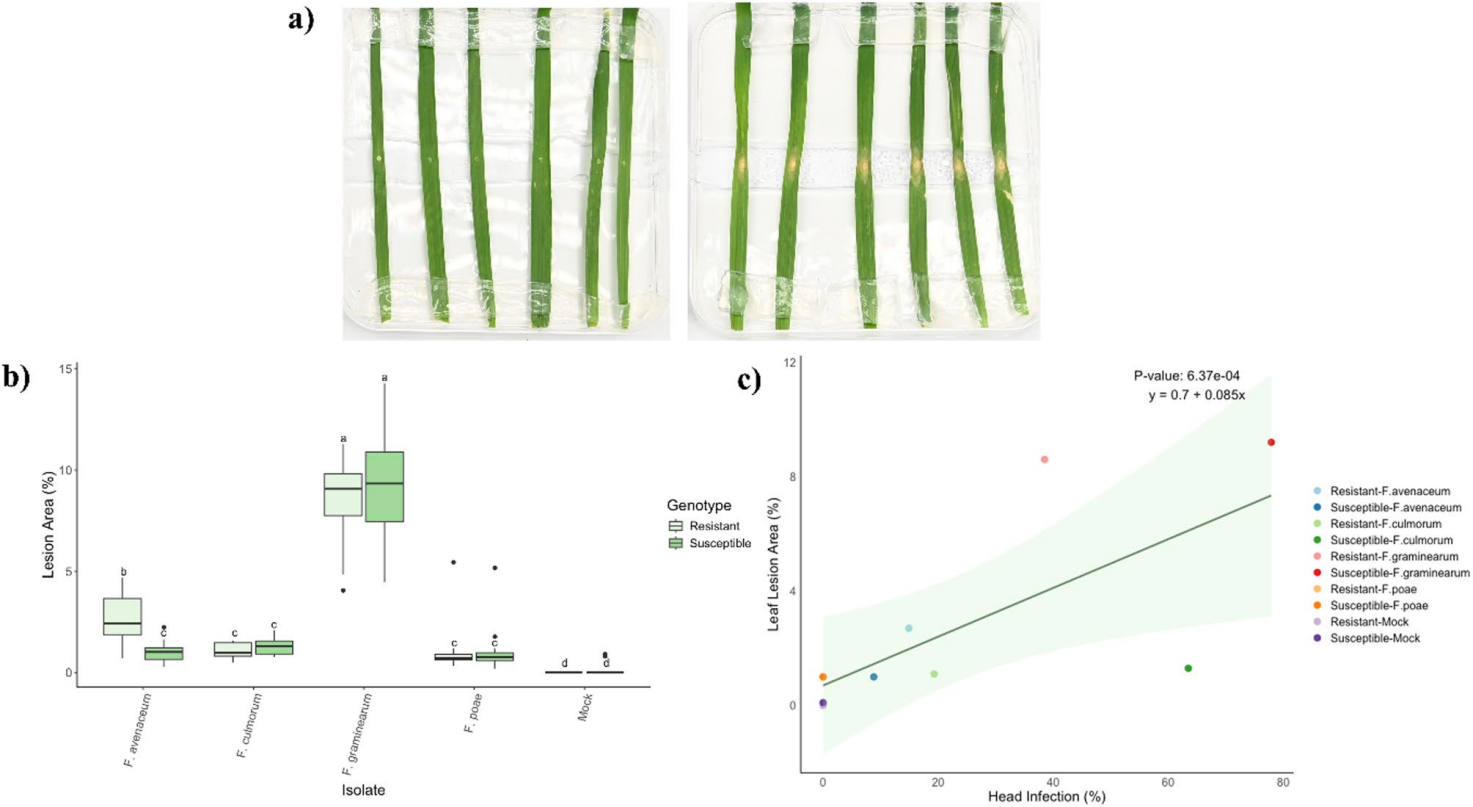
Lesions on the leaves in the detached leaf infection assay were measured three days post-inoculation. Wheat leaves inoculated with sterile water (control, left) are compared to leaves inoculated with *F. graminearum* (right) in the susceptible wheat cultivar. **a)** A representative image of the setup used for assessing fungal infection. Wheat leaves were placed on water-agar media, followed by inoculation with spore suspensions of different *Fusarium* species. **b)** Effect of *Fusarium* species treatment on lesion area (%) on leaves of resistant (3B5A) and susceptible (bbaa) wheat. Different letters above each boxplot indicate statistically significant (α = 0.05) differences in group means as determined by the Tukey HSD Post-hoc test (*n* = 18). **c)** Scatterplot comparing leaf lesion in leaf assay to head infection assay. The regression line indicates a significant correlation (*P* = 6.37E-04), with the shaded area representing the 95% confidence interval

There was an interaction between wheat genotype and *Fusarium* species treatments on leaf lesion area (*P =* 0.001; Table S13), thus the simple effects were analyzed. Similar to what was observed in other assays, leaves inoculated with *F. graminearum* had the highest percent lesion area (~ 9.0%) (*P* < 0.001; Fig. 3a, b). Interestingly, the resistant wheat genotype only showed a significant difference in lesion area compared to the susceptible genotype when inoculated with *F. avenaceum* (*P* < 0.001). This could suggest that *F. avenaceum* is more sensitive to the host genetic resistance mechanisms in leaf tissue, unlike *F. graminearum*, which seems to be more capable of overcoming these defenses. However, the variability observed in infection caused by different *Fusarium* species in the leaf assay may also be influenced by the limitations of using excised plant tissues [37]. Consistently, the leaf assay correlated the least with head infections across the *Fusarium* species (*P* = 6.37E-04; Fig. 3c).

### Head infection assay

**Fig. 4:**
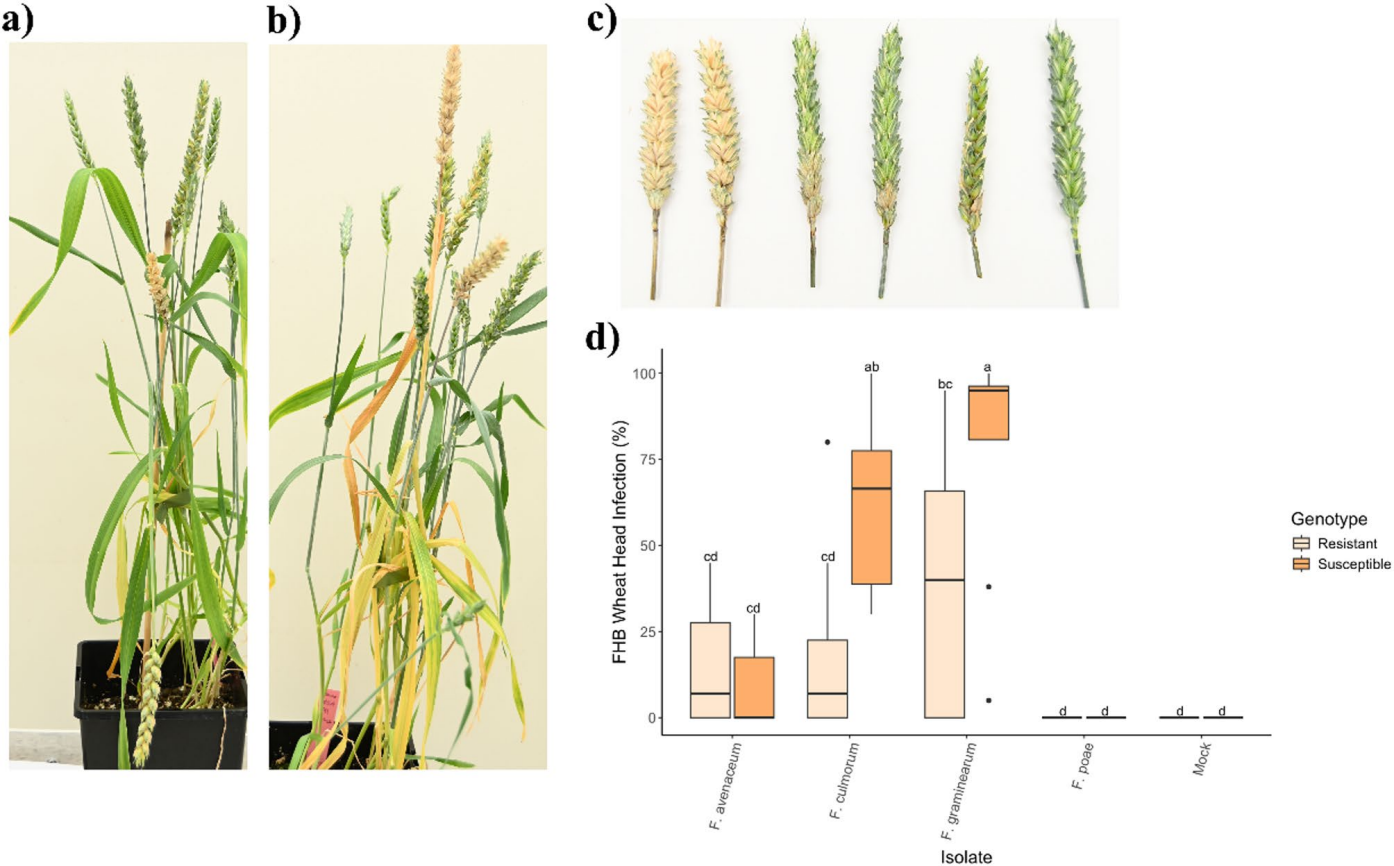
Infection assessment on spikes were measured at 10 days post inoculation with a head assay. Spikes of (**a**) resistant (3B5A) and (**b**) susceptible (bbaa) wheat cultivars were inoculated with spore suspensions of various *Fusarium* species to evaluate pathogen impact and cultivar response. (**c**) Disease symptoms were evaluated by assessing the extent of bleaching and necrosis on the spikelet. (**d**) Effect of *Fusarium* species treatment on head infection rate (%) on resistant and susceptible wheat. Different letters above each boxplot indicate statistically significant (α = 0.05) differences in group means as determined by the Tukey HSD Post-hoc test (*n* = 8)

For the head infection assay, there was a significant interaction between wheat genotype and *Fusarium* species treatments on infection rate (*P* = 0.001; Table S14), thus the simple effects were analyzed Specifically, while *F. graminearum* and *F. culmorum* exhibited high infection rates in both susceptible and resistant wheat spikes (Fig. 4a-c), *F. avenaceum* showed lower disease severity in the susceptible wheat compared to the other *Fusarium* species, while spikes inoculated with *F. poae* showed no symptoms (Fig. 4d). Furthermore, the susceptible wheat spikes infected with *F. graminearum* and *F. culmorum* had significantly higher infection rates compared to the resistant wheat infected with the same *Fusarium* isolates (*P* ≤ 0.017). This indicates that *F. graminearum* and *F. culmorum* are highly virulent on wheat heads, and the resistance of wheat significantly affects the infection rates for these species (Fig. 4d). In addition, the results for *F. graminearum* were highly variable, with infection rates in resistant wheat ranging from 0 to nearly 90%. Furthermore, several replicates of resistant wheat showed no infection rates when inoculated with *F. avenaceum* and *F. culmorum*. This significant variability from the other disease assays suggests that environmental or genetic factors could influence the wheat response to different *Fusarium* species.

## Discussion

Global shifts in the distribution of *Fusarium* species responsible for FHB highlight the importance of understanding FHB community dynamics and identifying resistant host germplasm [8]. Furthermore, recent population shift raises questions about the role of ecological variables mediating host-pathogen interactions. Understanding the interaction among dozens of FHB-causing *Fusarium* species, their hosts, and different environmental variables poses a significant challenge for current standard screening methodologies. In this study, we approach this challenge by aiming to identify high-throughput phenotyping methods capable of differentiating between FHB species and host genotypes while maintaining comparability to traditional head infection methods.

Among the high-throughput assays tested [23, 30, 33], seedling and coleoptile infections proved particularly effective. These methods provided rapid and reliable evaluations of wheat responses to different *Fusarium* species, offering a fast and straightforward alternative to traditional field trials. The head infection assay, widely used in greenhouse FHB studies, typically involves point inoculation inside florets, providing controlled conditions for assessing fungal virulence [35, 38]. Mesterhazy et al. [15] suggest that spray inoculation may better replicate field conditions by covering multiple resistance mechanisms. While spray inoculation is useful for large-scale screening, point inoculation remains essential when the goal is to precisely dissect specific resistance traits (e.g., the ability to spread through the rachis) with minimal external variability [29, 38]. However, head assays, whether by point or spray inoculation, often show inconsistent predictive value for field outcomes due to environmental variability, leading to experimental errors [39]. They are also resource-intensive, requiring weeks to months for plants to reach the heading stage, increasing time and costs [40, 41]. While head assays offer integral data about FHB development, our study highlights the potential advantages of coleoptile and seedling assays as faster, reliable alternatives for assessing *Fusarium* virulence in wheat. These assays may allow for prescreening of samples, reducing the extent of field trials, and for some experiments may be sufficient in themselves. Both assays showed strong comparability to the head infection assay, suggesting they can serve as effective early-stage phenotyping tools. Notably, both of these high-throughput assays provide results within 10 to 14 days, making them highly suitable for fast and large-scale screening efforts [30]. While the seedling assay is slightly more resource-intensive than coleoptile assay due to the space and time required for plant growth and inoculation, it provides valuable data on growth parameters such as germination rate, shoot length, root length, and disease severity [42]. Furthermore, these assays can be conducted under controlled environmental conditions, enhancing their utility and consistency [25, 30]. While head infection assays can also be performed under controlled conditions, the high-throughput assays in this study offer greater efficiency and reliability for screening a large number of *Fusarium* isolates and wheat genotypes.

The detached leaf assay, while efficient for large-scale screening, exhibited limitations in fully replicating the resistance dynamics observed in head infections. Detached tissue bioassays, as previously reported [37, 43], may alter natural resistance responses, leading to skewed pathogenicity assessments. This was evident in our study, where detached leaves failed to accurately represent the full wheat-*Fusarium* interaction. It was reported that excised tissues inoculated with *Pythium*, a soil-born pathogen, showed a reduction in natural resistance, sometimes giving the false impression that non-pathogenic isolates were pathogenic. This underscores the importance of complementing detached leaf assays with other methods that better mimic natural infection conditions [37].

Although both coleoptile and seedling assays provide valuable alternatives for early-stage, large-scale screening of wheat genotypes against *Fusarium* species, the head infection assay remains essential for comprehensive FHB evaluations. The ability of head assay to assess disease progression and mycotoxin accumulation under conditions mimicking natural field environments provides crucial insights into disease impact across diverse environmental and genetic contexts [44]. Furthermore, mycotoxin contamination at the head stage could serve as an early indicator for seed contamination [45–47], highlighting the potential predictive value of such assays for assessing downstream risks to grain safety. Trichothecene mycotoxins, particularly deoxynivalenol (DON), produced by *Fusarium* species such as *F. graminearum* and *F. culmorum*, are important for the progression of FHB and themselves pose significant threats to food safety. While not required for initial infection, DON is important for subsequent spread through the host [45]. Quantitative differences in the production of DON do not always correlate with virulence [48]. While disease severity in later-stage assays, such as head assays, is sometimes correlated with mycotoxin levels, the predictive power of DON production at earlier stages is less well understood and varies depending on genotype, fungal species, and environmental conditions [15]. As our study was aimed at understanding the role of *Fusarium* species and host resistance on virulence, we did not measure trichothecene production. However, our results showing differences in virulence emphasize the opportunity to help disentangle the importance of quantitative differences in toxin production across pathogens and host stages in future studies.

Variation in the virulence of different *Fusarium* species plays a significant role in shaping disease dynamics and informing effective management strategies. The differing virulence profiles of these species can influence disease outcomes and impact crop yield and quality, making it essential to evaluate their behavior across various host tissues [5, 49]. In this study, *F. graminearum* emerged as the most virulent pathogen across all assays, causing severe disease in multiple tissues, while *F. poae* exhibited lower virulence, consistent with previous literature identifying *F. graminearum* as a major cause of FHB. While *F. poae* is quite prevalent, this species generally contributes to less severe disease symptoms [1, 5, 7, 8, 10]. However, *F. poae* is known to produce nivelanol, trichothecene that is more cytotoxic than DON in some bioassays [1, 5, 7, 8, 10]. Its frequent co-occurrence with more aggressive *Fusarium* species compounds mycotoxin contamination risks, underscoring its significance in the FHB complex [50]. While our head infection assay detected minimal disease caused by *F. poae*, alternative high-throughput methods identified mild symptoms, highlighting their potential for studying less virulent pathogens and their interactions across different wheat tissues [51]. Our isolates of *F. culmorum* and *F. avenaceum* showed variable virulence depending on the plant tissue type, further emphasizing the potential for complex interactions between *Fusarium* species and host tissues. Such complexity warrants further investigation to comprehensively understand the dynamics of infection and mycotoxin production across different genotypes and under various growth conditions.

Infections in plant tissues beyond the head, such as leaves, stems, and seedlings, are important for understanding the full scope of FHB-pathogen’s lifecycle and disease dynamics. *Fusarium* species are capable of surviving in plant residues and infected tissues, where they can overwinter and serve as reservoirs for the fungus, contributing to inoculum loads in subsequent growing seasons [5, 52]. The virulence of *F. avenaceum* and *F. culmorum* varied across assays in our study, suggesting possible distinct infection strategies and tissue-specific virulence factors. While these species are known to be associated with seedling blights, root and crown rots [53], further studies are needed to explicitly link these findings to those disease symptoms. While most FHB resistance research focuses on head infections, recent studies highlight the importance of considering the entire plant in disease management. Fungal colonization in non-head tissues, such as stalks and leaves, may act as primary inoculum sources for secondary infections in the head, either through direct spread or via the production of airborne conidia [54]. Beyond their role in FHB, *F. pseudograminearum*, *F. culmorum*, and *F. graminearum* are also major causal agents of Fusarium crown rot (FCR), which affects wheat crowns and roots and can significantly impact yield [55]. Importantly, both FHB and FCR infected plant residue can serve as a primary inoculum source for both diseases under favorable conditions, highlighting the need for an integrated approach to *Fusarium* disease management [55, 56]. This suggests the necessity of broadening the scope of breeding programs to not only target head resistance but also to enhance resistance across all plant tissues. This broader approach will help reduce pathogen reservoirs and limit the spread of inoculum throughout the growing season, contributing to more effective disease management strategies.

Environmental factors, particularly temperature and humidity, play a crucial role in *Fusarium* pathogenicity and mycotoxin production [57, 58]. In Europe, *F. culmorum* and *F. avenaceum* dominate in cooler regions, while *F. graminearum* is more prevalent in warmer climates with higher virulence and toxin production [57]. These variations must be carefully considered when interpreting phenotyping assay results, as they can significantly impact the accuracy of screening methods for *Fusarium* resistance in wheat. Our efforts enable future large-scale high-throughput efforts that can begin to address the interplay of these important variables.

Breeding efforts for FHB resistance have made significant progress, but the complexity of FHB resistance, influenced by both host genetics and pathogen variability, continues to challenge breeding programs. Research on *F. graminearum* has revealed a complex and diverse set of resistance mechanisms [1, 10, 17]. Some studies have suggested that FHB resistance in wheat is likely non-specific, meaning that resistance to *F. graminearum* often confers protection against other *Fusarium* species [15, 59, 60]. Our study supports this concept, as we observed that isogenic wheat lines carrying well-characterized resistance loci, including *Fhb1* (chromosome 3B) and *Qfhs.ifa-5 A* (chromosome 5 A), not only provided resistance to *F. graminearum*, but also to other *Fusarium* species. This is consistent with the idea that high resistance to one species is often associated with resistance to other species [15]. This broad resistance is crucial for long-term FHB control. However, the variability in virulence and potential multi-toxin contamination complicates breeding efforts, emphasizing the need for further research and improved strategies integrating disease resistance with food safety [15, 48]. Our findings highlight the importance of considering interspecies interactions in screening efforts to improve the robustness of resistance to different *Fusarium* species [11, 15]. However, this study only assessed the virulence of four *Fusarium* species but did not account for variability in aggressiveness between isolates or inoculum concentrations, which are crucial aspects, as highlighted by Mesterházy, 2024 [15]. While our results are consistent with previous inferences of species virulence, our focus was to establish the potential usefulness of high-throughput methodologies. Future studies should incorporate multiple isolates and controlled conditions to better understand species- and isolate-specific virulence patterns.

Overall, this study evaluated the effectiveness of high-throughput phenotyping methods in distinguishing wheat responses to different *Fusarium* species. The seedling and coleoptile assays, in particular, proved to be fast and efficient, offering strong comparability to traditional head infection assays, while enabling more rapid data collection. This ability to quickly and accurately assess phenotypic variation and genotype-specific responses is crucial for advancing large-scale screenings and improving the efficiency of resistance breeding programs. Since our study focused on just two genotypes, future research should evaluate high-throughput phenotyping methods across a broader range of wheat cultivars, including those with moderate resistance or susceptibility.

## Conclusion

This study demonstrates that high-throughput phenotyping assays, such as coleoptile and seedling assays are effective alternatives to traditional head infection assays for large-scale screening of fungal virulence and wheat FHB resistance. These methods also address many limitations of conventional methods, offering significant advantages in speed, cost, and space efficiency while providing valuable insights into interactions among different *Fusarium* species. However, the traditional head assay remains essential for assessing disease progression and mycotoxin accumulation under field conditions. Future research should focus on integrating high-throughput assays with field evaluations to create a complete framework for FHB resistance breeding. Such an approach will not only streamline the identification of resistant genotypes but also deepen our understanding of the complex interactions between wheat and *Fusarium* species.

## Electronic supplementary material

Below is the link to the electronic supplementary material.

Supplementary Material 1

## Data Availability

The data supporting the findings of this study are available from the corresponding authors upon request.
