# Supplementary material for: Comparative analysis of distinct phenotyping methods for assessing wheat resistance and pathogen virulence among Fusarium species causing head blight disease
Source: Plant Methods. 2025 Jun 16;21:85. doi: 10.1186/s13007-025-01402-8 (PMC12168331; doi:10.1186/s13007-025-01402-8)
Supplement: Supplementary file 1 — Supplementary Material 1 [file 13007_2025_1402_MOESM1_ESM.docx]

**Supplemental Figures and Tables**

**
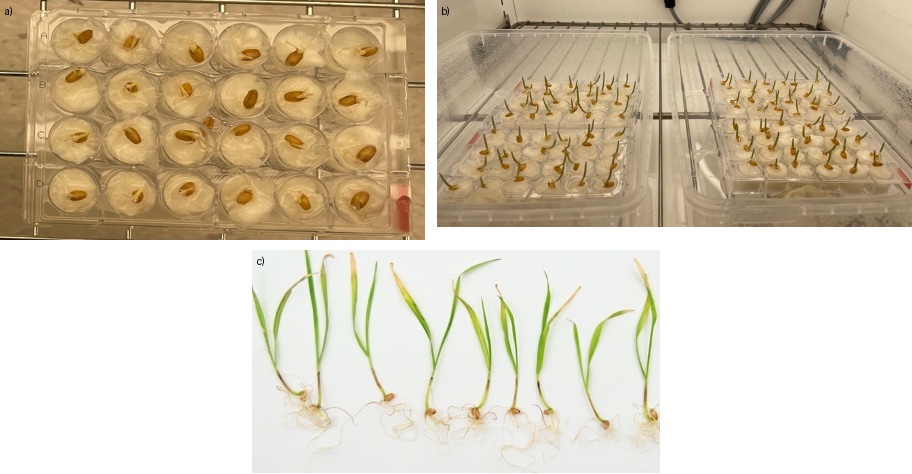
**

**Supplementary Figure 1.** The experimental setup used to evaluate coleoptile infection**. a)** Seeds were germinated, and the coleoptiles were placed on 24-well plates for infection assays. **b)** The tips of three-day old coleoptiles were cut and was infected with different *Fusarium* species.

**
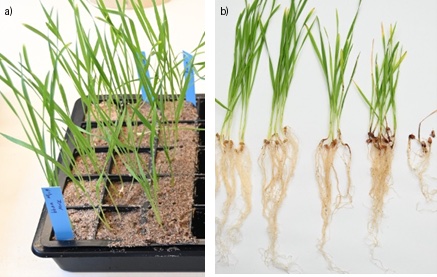
**

**Supplementary Figure 2.** **a**) Assessment of *Fusarium* species virulence in seedling infection assay: Agar blocks of fungal species were used to inoculate seeds of susceptible and resistant wheats. **b)** Symptoms on stem and root (disease index), as well as shoot and root length, were measured 14 days post inoculation to evaluate fungal virulence.


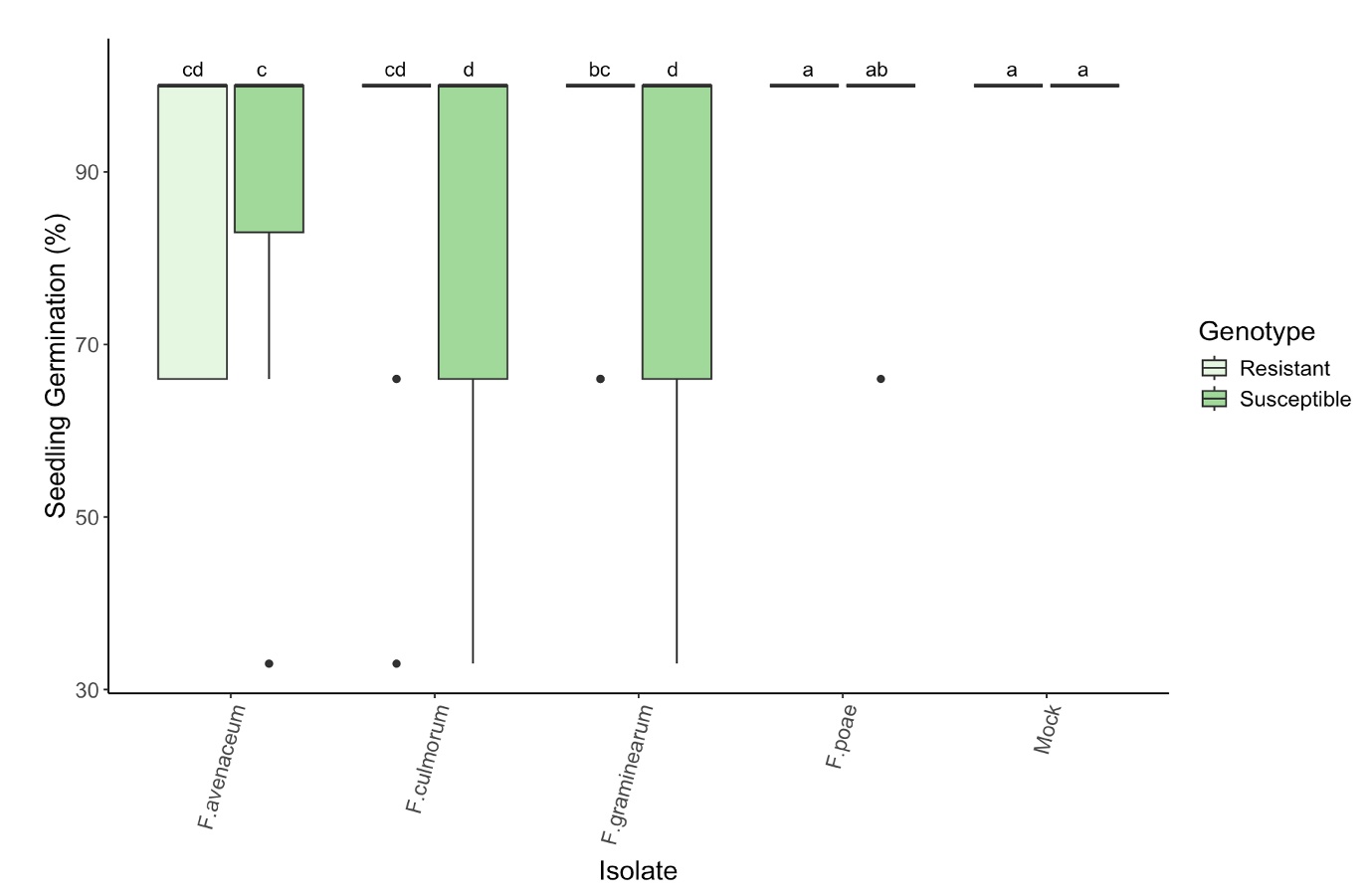


**Supplementary Figure 3.** Effect of *Fusarium* species treatment on seedling germination (%) on resistant (3B5A) and susceptible (bbaa) wheat 14 days post inoculation. Different letters above each boxplot indicate statistically significant (α = 0.05) differences in group means as determined by the Tukey HSD Post-hoc test (n = 15).


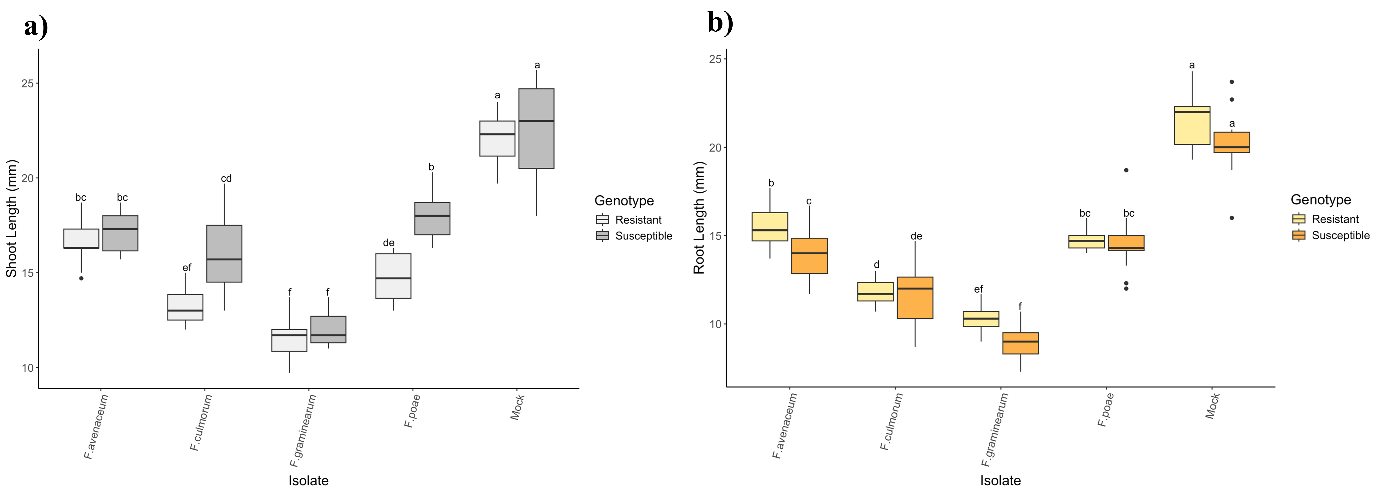


**Supplementary Figure 4.** Effect of *Fusarium* species treatment on (**a**) shoot length (mm) and (**b**) root length (mm) on resistant (3B5A) and susceptible (bbaa) wheat for the seedling assay 14 days post inoculation. Different letters above each boxplot indicate statistically significant (α = 0.05) differences in group means as determined by the Tukey HSD Post-hoc test (n = 15).


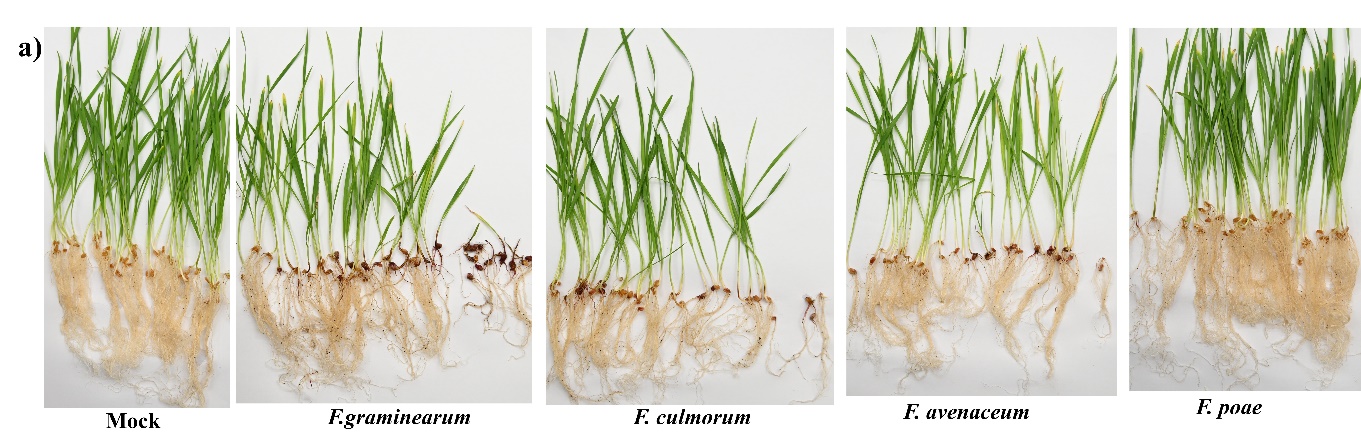


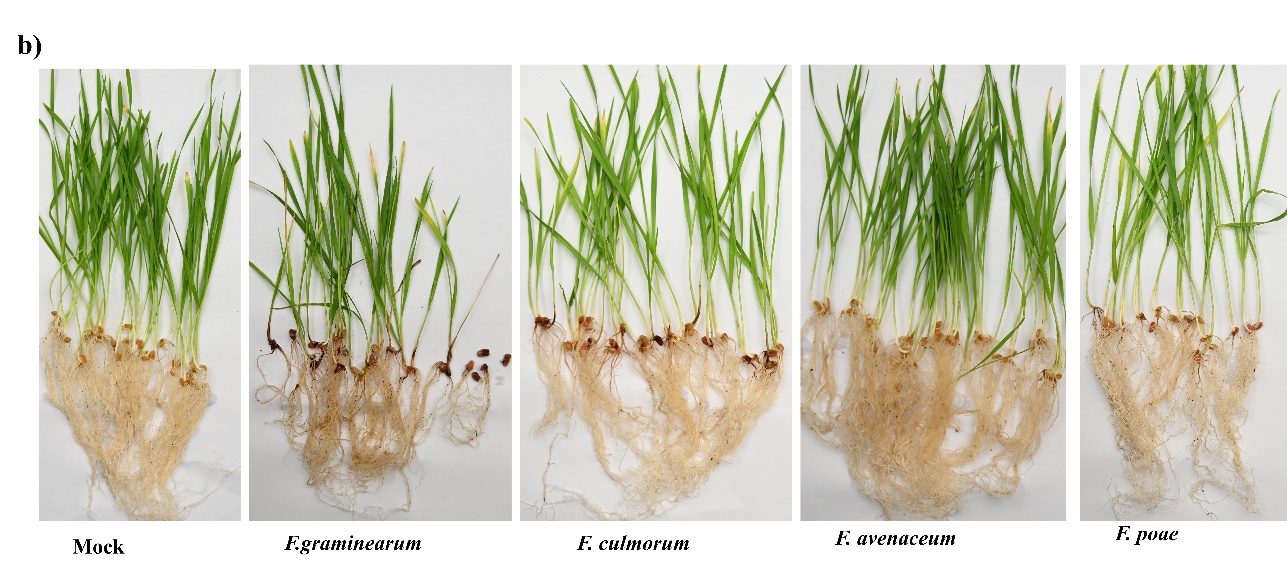


**Supplementary Figure 5. a)** Symptoms of FHB on seedlings of the susceptible cultivar (bbaa) caused by various *Fusarium* species. **b)** Symptoms of FHB on seedlings of the resistant cultivar (3B5A) caused by various *Fusarium* species**.**

**ANOVA tables for *Fusarium* infections across wheat tissues and assays:**

**Supplementary Table 1.** Whole ANOVA table on mean rank-normalized data. Within each assay measurement, the data were rank normalized to compare measurements across different assays.

| **Response: Rank-normalized disease measurements** | **Sum Sq** | **Df** | **F value** | **P value** |
| --- | --- | --- | --- | --- |
| Genotype | 288 | 1 | 1.005 | 0.316 |
| Isolate | 309698 | 4 | 270.435 | < 2.2 e-16 |
| Assay | 18737 | 3 | 21.814 | 1.033 e-13 |
| Genotype*Isolate | 2955 | 4 | 2.580 | 0.0359 |
| Genotype*Assay | 2040 | 3 | 2.374 | 0.0685 |
| Isolate*Assay | 32650 | 12 | 9.503 | < 2.2 e-16 |
| Genotype*Isolate*Assay | 7318 | 12 | 2.130 | 0.013 |
| Residuals | 303473 | 1060 |  |  |

**Supplementary Table 2.** *Fusarium graminearum* ANOVA table on mean rank-normalized data (ANOVA for data represented in Table 2).

| **Response: Rank-normalized disease measurements** | **Sum Sq** | **Df** | **F value** | **P value** |
| --- | --- | --- | --- | --- |
| Genotype | 2486.3 | 1 | 19.231 | 1.842 e-05 |
| Assay | 2250.1 | 3 | 5.8013 | 0.0007 |
| Genotype*Assay | 877.5 | 3 | 2.262 | 0.082 |
| Residuals | 27408.3 | 212 |  |  |

**Supplementary Table 3.** *Fusarium culmorum* ANOVA table on mean rank-normalized data (ANOVA for data represented in Table 2).

| **Response: Rank-normalized disease measurements** | **Sum Sq** | **Df** | **F value** | **P value** |
| --- | --- | --- | --- | --- |
| Genotype | 236 | 1 | 0.972 | 0.325 |
| Assay | 13156 | 3 | 18.058 | 1.789 e-10 |
| Genotype*Assay | 2923 | 3 | 4.012 | 0.008 |
| Residuals | 51484 | 212 |  |  |

**Supplementary Table 4.** *Fusarium avenaceum* ANOVA table on mean rank-normalized data (ANOVA for data represented in Table 2).

| **Response: Rank-normalized disease measurements** | **Sum Sq** | **Df** | **F value** | **P value** |
| --- | --- | --- | --- | --- |
| Genotype | 404 | 1 | 1.102 | 0.294 |
| Assay | 7440 | 3 | 6.772 | 0.0002 |
| Genotype*Assay | 5060 | 3 | 4.606 | 0.003 |
| Residuals | 77637 | 212 |  |  |

**Supplementary Table 5.** *Fusarium poae* ANOVA table on mean rank-normalized data (ANOVA for data represented in Table 2).

| **Response: Rank-normalized disease measurements** | **Sum Sq** | **Df** | **F value** | **P value** |
| --- | --- | --- | --- | --- |
| Genotype | 107 | 1 | 0.315 | 0.553 |
| Assay | 9466 | 3 | 10.361 | 2.161 e-06 |
| Genotype*Assay | 446 | 3 | 0.4885 | 0.690 |
| Residuals | 64560 | 212 |  |  |

**Supplementary Table 6.** Mock ANOVA table on mean rank-normalized data (ANOVA for data represented in Table 2).

| **Response: Rank-normalized disease measurements** | **Sum Sq** | **Df** | **F value** | **P value** |
| --- | --- | --- | --- | --- |
| Genotype | 10 | 1 | 0.0255 | 0.873 |
| Assay | 19075 | 3 | 16.361 | 1.333 e-09 |
| Genotype*Assay | 51 | 3 | 0.0434 | 0.987 |
| Residuals | 82385 | 212 |  |  |

**Supplementary Table 7.** Coleoptile Assay: Stem Lesion ANOVA table

| **Response: Stem Lesion (mm)** | **Sum Sq** | **Df** | **F value** | **P value** |
| --- | --- | --- | --- | --- |
| Genotype | 0.41 | 1 | 0.039 | 0.842 |
| Isolate | 1477.00 | 4 | 35.720 | < 2e-16 |
| Genotype*Isolate | 35.63 | 4 | 0.861 | 0.489 |
| Residuals | 1137.08 | 110 |  |  |

**Supplementary Table 8.** Coleoptile Assay: Leaf Lesion ANOVA table

| **Response: Leaf Lesion (mm)** | **Sum Sq** | **Df** | **F value** | **P value** |
| --- | --- | --- | --- | --- |
| Genotype | 15996.9 | 1 | 47.507 | < 2 e-16 |
| Isolate | 1771.0 | 4 | 21.038 | 1.999 e-05 |
| Genotype*Isolate | 1606.3 | 4 | 4.770 | 0.001 |
| Residuals | 9259.9 | 110 |  |  |

**Supplementary Table 9.** Seedling Assay: Germination Rate ANOVA table

| **Response: Germination Rate (%)** | **Sum Sq** | **Df** | **F value** | **P value** |
| --- | --- | --- | --- | --- |
| Genotype | 482 | 1 | 1.951 | 0.164 |
| Isolate | 4469 | 4 | 4.520 | 0.001 |
| Genotype*Isolate | 504 | 4 | 0.509 | 0.728 |
| Residuals | 34603 | 140 |  |  |

**Supplementary Table 10.** Seedling Assay: Shoot Length ANOVA table

| **Response: Shoot Length (mm)** | **Sum Sq** | **Df** | **F value** | **P value** |
| --- | --- | --- | --- | --- |
| Genotype | 86.94 | 1 | 42.016 | 1.439 e-09 |
| Isolate | 1768.97 | 4 | 213.716 | < 2.2 e-16 |
| Genotype*Isolate | 61.54 | 4 | 7.434 | 1.849 e-05 |
| Residuals | 289.70 | 140 |  |  |

**Supplementary Table 11.** Seedling Assay: Root Length ANOVA table

| **Response: Root Length (mm)** | **Sum Sq** | **Df** | **F value** | **P value** |
| --- | --- | --- | --- | --- |
| Genotype | 33.99 | 1 | 21.410 | 8.365 e-06 |
| Isolate | 2146.97 | 4 | 338.126 | < 2.2 e-16 |
| Genotype*Isolate | 14.38 | 4 | 2.264 | 0.065 |
| Residuals | 222.24 | 140 |  |  |

**Supplementary Table 12.** Seedling Assay: Disease Index ANOVA table

| **Response: Disease Index** | **Sum Sq** | **Df** | **F value** | **P value** |
| --- | --- | --- | --- | --- |
| Genotype | 6.092 | 1 | 26.260 | 9.716 e-07 |
| Isolate | 154.404 | 4 | 166.389 | < 2.2 e-16 |
| Genotype*Isolate | 4.462 | 4 | 4.897 | 0.001 |
| Residuals | 32.479 | 140 |  |  |

**Supplementary Table 13.** Leaf Assay ANOVA table

| **Response: Leaf lesion Area (%)** | **Sum Sq** | **Df** | **F value** | **P value** |
| --- | --- | --- | --- | --- |
| Genotype | 0.194 | 1 | 2.426 | 0.121 |
| Isolate | 111.694 | 4 | 349.653 | < 2 e-16 |
| Genotype*Isolate | 2.526 | 4 | 7.908 | 7.188 e-06 |
| Residuals | 13.576 | 170 |  |  |

**Supplementary Table 14.** Head Assay ANOVA table

| **Response: Head Infection (%)** | **Sum Sq** | **Df** | **F value** | **P value** |
| --- | --- | --- | --- | --- |
| Genotype | 4805 | 1 | 10.237 | 0.002 |
| Isolate | 44101 | 4 | 23.490 | 2.431 e-12 |
| Genotype*Isolate | 9379 | 4 | 4.995 | 0.001 |
| Residuals | 32855 | 70 |  |  |
